# Distinct Potentially Adaptive Accumulation of Truncation Mutations in Salmonella enterica serovar Typhi and Salmonella enterica serovar Paratyphi A

**DOI:** 10.1128/spectrum.01969-21

**Published:** 2022-05-04

**Authors:** Stephy Mol Robinson, Vyshakh Rajachandran, Suchismita Majumdar, Satabdi Saha, Sneha Das, Sujay Chattopadhyay

**Affiliations:** a Centre for Health Science and Technology, JIS Institute of Advanced Studies and Research Kolkata, JIS University, Kolkata, West Bengal, India; Health Canada

**Keywords:** truncation mutation, premature stop codon, adaptive evolution, convergent mutation, *Salmonella* Typhi, *Salmonella* Paratyphi A, truncation mutation

## Abstract

Gene inactivation through the accumulation of truncation (or premature stop codon) mutations is a common mode of evolution in bacteria. It is frequently believed to result from reductive evolutionary processes allowing purging of superfluous traits. However, several works have demonstrated that, similar to the occurrences of inactivating nonsynonymous (i.e., amino acid replacement) mutations under positive selection pressures, truncation mutations can also be adaptive where specific traits deleterious in particular environmental conditions need to be inactivated for survival. Here, we performed a comparative analysis of genome-wide accumulation of truncation mutations in Salmonella enterica serovar Typhi and Salmonella enterica serovar Paratyphi A. Considering the known convergent evolutionary trajectories in these two serovars, we expected a strong overlap of truncated genes in *S*. Typhi and *S.* Paratyphi A, emerging through either reductive or adaptive dynamics. However, we detected a distinct set of core truncated genes encoding different overrepresented functional clusters in each serovar. In 54% and 28% truncated genes in *S*. Typhi and *S.* Paratyphi A, respectively, inactivating mutations were acquired by only different subsets of isolates, instead of all isolates analyzed for that serovar. Importantly, 62% truncated genes (*P* < 0.001) in *S.* Typhi and *S.* Paratyphi A were also targeted by convergent amino acid mutations in different serovars, suggesting those genes to be under selection pressures. Our findings indicate significant presence of potentially adaptive truncation mutations in conjunction with the ones emerging due to reductive evolution. Further experimental and large-scale bioinformatic studies are necessary to better explore the impact of such adaptive footprints of truncation mutations in the evolution of bacterial virulence.

**IMPORTANCE** Detecting the adaptive mutations leading to gene inactivation or loss of function is crucial for understanding their contribution in the evolution of bacterial virulence and antibiotic resistance. Such inactivating mutations, apart from being of nonsynonymous (i.e., amino acid replacement) nature, can also be truncation mutations, abruptly trimming the length of encoded proteins. Importantly, the notion of reductive evolutionary dynamics is primarily accepted toward the accumulation of truncation mutations. However, our case study on *S.* Typhi and *S.* Paratyphi A, two human-restricted systemically invasive pathogens exerting similar clinical manifestations, indicated that a significant proportion of truncation mutations emerge from positive selection pressures. The candidate genes from our study will enable directed functional assays for deciphering the adaptive role of truncation mutations in *S.* Typhi and *S.* Paratyphi A pathogenesis. Also, our genome-level analytical approach will pave the way to understand the contribution of truncation mutations in the adaptive evolution of other bacterial pathogens.

## INTRODUCTION

Loss-of-function mutations represent a common, yet understudied, mechanism of bacterial evolution (1). This leads to inactivation of gene functions, thereby forming pseudogenes. Gene inactivation can happen via a frameshift mutation, a truncation mutation (i.e., a premature nonsense or stop codon mutation in presence or absence of frameshift mutation), a missense mutation, or insertion of transposable elements. Several works, especially in enteric bacteria, have been performed toward a systematic search of inactivated genes across the genomes (2–6). It is generally believed, particularly in obligate symbiotic bacterial pathogens, that pseudogenization results from reductive evolution following “use-or-lose” dynamics to allow purging of traits since the genes being inactivated are of no use in the organism owing to the organism’s lifestyle and utilization of host genome machineries (7–9). However, there are reports suggesting that the truncation mutations (TnM) leading to gene inactivation (and often followed by gene deletion over evolutionary time) can also happen under adaptive pressures as part of “die-or-lose” dynamics where specific traits detrimental in particular environmental conditions are to be inactivated for immediate adaptive advantage and survival (10–17).

Two systemically invasive human-restricted serovars of Salmonella enterica subspecies I, Salmonella enterica serovar Typhi and Salmonella enterica serovar Paratyphi A, represent a good model to study pseudogene formation and gene deletion in comparison with several self-limiting gastroenteritis-causing serovars, such as Salmonella enterica serovar Typhimurium, which are adapted to a broad range of hosts (18, 19). Typhi and Paratyphi A, as expected for their adaptation to the same niche with similar modes of pathogenesis, have been shown to undergo adaptive convergent evolution through both recombinational and mutational processes (20, 21). Previous work has also showed the formation of pseudogenes to converge in the course of evolution of these two serovars (22). Interestingly, any such convergence in the emergence of pseudogenes could be attributed to either reductive evolution because of the host-restricted lifestyle or adaptive evolution where specific selection pressures act on those genes to be inactivated through parallel evolutionary trajectories in two serovars. In this regard, a comparative genomic analysis of the accumulation of TnM, with an emphasis on these two serovars, has long been due. Our study here identified the genome-wide truncated genes of Typhi and Paratyphi A, resulting exclusively from premature stop codons in the absence of frameshift mutations. We compared this set in the background of all core genes (i.e., the genes present in all analyzed isolates) in a set of 214 publicly available completely sequenced genomes from these two serovars plus Salmonella enterica serovar Gallinarum, Salmonella enterica serovar Pullorum, and Salmonella enterica serovar Typhimurium. While Gallinarum and Pullorum represent poultry-restricted serovars causing systemically invasive infections, Typhimurium can infect a broad range of hosts causing primarily self-limiting gastroenteritis apart from occasional invasive infections. We selected such a wide range of serovar isolates apart from the Typhi and Paratyphi genomes in order to identify the core fraction of protein-coding genes which would not be restricted to only these two serovars but rather be a representative set of a larger group of serovars irrespective of host restriction (human versus poultry) or infection type (systemically invasive versus noninvasive) and then to estimate the dynamics of truncation mutation evolution in Typhi and Paratyphi A. This would result in the detection of truncated genes in Typhi and/or Paratyphi A serovars distinct from those found also in the invasive isolates of Typhimurium or other host-restricted serovars or in the Typhimurium isolates causing self-limiting gastroenteritis.

We anticipated that, in the core fraction of genomes from diverse *Salmonella* serovars, the sets of genes acquiring TnM will converge in Typhi and Paratyphi A, as demonstrated earlier (22). An earlier study reported that the pseudogenization of *sopA* and *sopE2* contributed to the virulence of Typhi (16). Another more recent work suggested footprints of positive selection in 84 inactivated genes relating to virulence, chemotaxis, biofilm formation, motility, and resistance to antibiotics and other toxic compounds in *Salmonella* (17). With this in view, we hypothesized that a considerable fraction of core genes would acquire TnM under positive selection pressures instead of reductive evolutionary pressures due to their superfluous nature. Therefore, we also analyzed genome-wide presence of convergent amino acid mutations that would potentially accumulate under adaptive pressures and mapped them with the set of truncated genes to detect the overlap, if any. We found significant overlap of the truncated genes and the genes with convergent mutations in different isolates/serovars. Also, to our surprise, we found significantly different sets of genes, and overrepresented protein functional clusters as well, accumulating TnM between Typhi and Paratyphi A. Moreover, differential presence of TnM in isolates of a single serovar indicates that localized selection pressures might be in action for the inactivation of many genes in the evolution of Typhi and Paratyphi A isolates.

## RESULTS AND DISCUSSION

### Two distinct clonal complexes represented by Typhi and Paratyphi A isolates.

In this study, completely sequenced genomes from 116 Typhi isolates and 6 Paratyphi A isolates were analyzed in the background of another set of 92 genomes, from 2 Gallinarum and 3 Pullorum isolates (both serovars being host-restricted and systemically invasive, similar to Typhi and Paratyphi A), and from 87 Typhimurium isolates (Table S1 found at https://figshare.com/articles/dataset/Supplemental_Material_FOR_publication_pdf/19656858). In contrast to the remaining serovars selected here, Typhimurium primarily infects a broad range of host species causing self-limiting noninvasive gastroenteritis, apart from occasional representation of isolates causing invasive nontyphoidal infection. Importantly, our set of Typhimurium genomes include at least 31 isolates which were mentioned to cause invasive nontyphoidal infections. We intended to extract the core protein-coding genes from this diverse set of serovars being restricted to humans or poultry or showing broad host-range and causing systemically invasive typhoidal/nontyphoidal as well as noninvasive infections.

We performed multilocus sequence typing (MLST) to estimate the clonal diversity of our data set (23, 24). Of all Typhi isolates, 75 isolates (65%) were found to represent sequence type (ST) 1, while there were four other STs, 2, 8, 2138, and 2209, represented by 36, 1, 1, and 3 isolates, respectively. Interestingly, these four STs were members of a single clonal complex (CC), CC2. On the other hand, there were two STs in our analyzed Paratyphi A isolates, ST85 and ST129, represented by 3 isolates each. Again, these two STs are also part of a single clonal complex, CC85. We calculated the pairwise nucleotide diversity (π) for the MLST loci within Typhi and Paratyphi A, as well as between these two serovars. As expected, the π values for the Typhi isolates (0.00011 ± 0.000102) were similar (*P* = 0.79) to those for the Paratyphi A isolates (0.00008 ± 0.00005). This suggests that the difference in sample size considered for the two serovars (116 for Typhi versus 6 for Paratyphi A) does not affect the intraserovar diversity. In contrast, these intraserovar diversity values were significantly lower (*P* < 0.02) than the value between Typhi and Paratyphi A (0.007 ± 0.003). This led to the expectation of an unbiased comparative analysis of the frequency of truncation and other mutations in these two serovars, even with drastically different sample sizes between serovars.

### Serovar-specific frequency of TnM genes found independent of the number of isolates analyzed.

Our comparative genomic analysis revealed a total of 3,368 core genes in the set of 214 genomes of Typhi, Paratyphi A, Gallinarum, Pullorum, and Typhimurium. Of these, 1,136 core genes accumulated TnM via premature stop codons in at least one of the analyzed serovars, and we call such genes here TnM genes. Although we restricted our analysis to the completely sequenced and annotated genomes, presence of premature stop codons in a gene might also randomly result from sequencing errors, and in such case, it is likely that the higher the number of isolates analyzed for a given serovar, the higher the number of TnM genes would be in that serovar. If sequencing errors were considered a major factor, there was a high possibility that it would be clearly reflected in our scenario, since the number of isolates we analyzed for 5 serovars ranged from as low as 2 to as high as 116.

**FIG 1:**
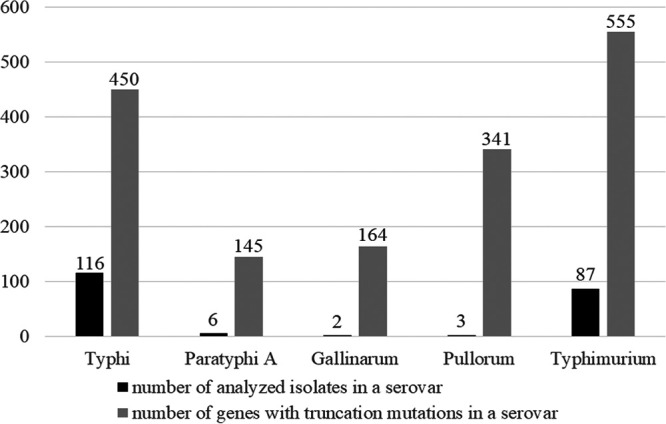
Comparative view of the number of isolates analyzed and the number of core genes with truncation mutations (TnM) in each serovar.

Interestingly, we detected the highest number of TnM genes, 555, in Typhimurium serovar with 87 isolates, while there were 450 TnM genes in Typhi with 116 isolates (Fig. 1). Also, Paratyphi A, represented by 6 isolates, showed only 145 TnM genes, in contrast to 164 and 341 TnM genes, respectively, in Gallinarum (with 2 isolates) and Pullorum (with 3 isolates). This indicated that the number of isolates analyzed for a given serovar did not considerably affect the frequency of TnM genes in that serovar, thereby minimizing the possibility of sequencing errors or of huge variation in serovar-specific sample size to be potential contributors to our results.

### Significant excess of mutational convergence in TnM genes showing negligible overlap between Typhi and Paratyphi A.

Accumulation of TnM leading to inactivation of genes, followed by pseudogenization, is primarily thought to result from reductive evolution because of the superfluous nature of such genes in the genome of the organism. However, as mentioned earlier, gene inactivation is a major mode of adaptive evolution in bacteria, and one common way for a gene to be inactivated is via accumulation of premature stop codons, i.e., truncation mutations. On the other hand, the repeated amino acid mutations that occur independently (i.e., in a phylogenetically unlinked way) at a particular position are known to happen under potential positive selection pressures, suggesting fitness advantage of such mutant variants, and are called convergent mutations (CM) (21, 25–30). While such mutational convergence suggests adaptive evolution, it is highly likely that many such convergent mutations are inactivating amino acid changes, thereby offering the same result as that of truncation mutations, as shown earlier for multiple *Salmonella* serovars (30). To our surprise, of 1,136 core TnM genes, we detected 609 genes (54%) to accumulate CM in the encoded amino acid sequences in some other isolates with intact genes, thereby suggesting the presence of selection pressures in the evolution of such genes in *Salmonella*. In contrast, the remaining 2,232 core genes in our data set (which did not show TnM in any alleles) included only 716 genes (32%) that accumulated CM, showing significant overlap of CM in TnM genes (Fisher’s exact test *P* < 0.0001).

As we concentrated on Typhi and Paratyphi A specifically, we found 160 genes that accumulated TnM in either or both of these serovars, exclusively or along with other serovars (Table S2 found at https://figshare.com/articles/dataset/Supplemental_Material_FOR_publication_pdf/19656858). Of this set, there were 71 (44.4%) genes where only half (≤50%) of the sequence length remained intact. In another 42 genes, TnM occurred somewhere between 50% and 75% of the length region, combining a set of 113 genes (71%) where at least one-fourth of the encoded protein would be missing if the genes were expressed to encode the proteins. This led us to assume that the TnM detected in our study primarily led to gene inactivation. Although TnM somewhere in the gene may also lead to neofunctionalization of the encoded protein, we can consider this to be inactivation with respect to original function of the full-length protein which gets lost. Similarly, there is always a possibility that the TnM toward the end of a gene may sometimes have enough disrupting effect to lead to inactivation.

**FIG 2:**
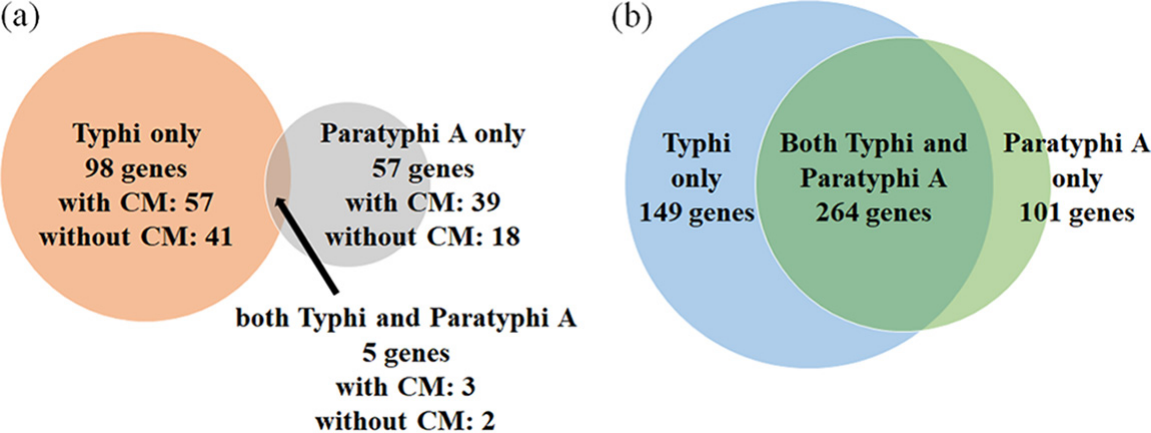
Venn diagrams showing (a) the frequency of core truncated genes (with and without convergent mutations) and (b) the frequency core nontruncated genes with convergent mutations in Typhi, Paratyphi A, or both. CM denotes convergent amino acid mutations.

Typhi and Paratyphi A, being restricted to human host and showing similar disease manifestation via similar modes of pathogenesis, are expected to develop common tricks to adapt to similar environmental conditions (31, 32). This resulted in convergent adaptive evolution in these two serovars, as demonstrated earlier to happen via both recombination and mutation (20, 21). Therefore, we next analyzed the overlap of TnM genes in Typhi and Paratyphi A. Any significant overlap, interestingly, could be explained through two possible mechanisms. Similar to recombinational and mutational adaptive convergence, there might be common adaptive pressures also on the inactivation of specific genes in both the serovars leading to the significant overlap of TnM genes. Alternatively, the significant overlap also might result from reductive evolution where the same set of genes became superfluous in these two serovars which had adapted and restricted themselves to infect the same host organs in a similar fashion. However, the overlap of TnM genes appeared to be limited (Fig. 2a). There were only 5 genes that accumulated TnM both in Typhi and Paratyphi, while 98 genes accumulated TnM in Typhi (80 exclusively in Typhi and 18 along with some other serovars but not Paratyphi A) and 57 genes accumulated TnM in Paratyphi A (45 exclusively in Paratyphi A and 12 along with some other serovars but not Typhi) (Table S2 found at https://figshare.com/articles/dataset/Supplemental_Material_FOR_publication_pdf/19656858). Such a scenario rules out any major impact of either common reductive evolution or adaptive convergence toward the accumulation TnM genes in these two human-restricted serovars.

In this set of 160 genes with TnM in Typhi, Paratyphi A, or both, similar to the overall set of TnM genes, we detected as many as 99 genes (62%) with CM (Table S2 found at https://figshare.com/articles/dataset/Supplemental_Material_FOR_publication_pdf/19656858). In contrast, there were 514 genes (16%) with CM in Typhi, Paratyphi A, or both in the set of 3,208 core genes that did not accumulate any TnM in these two serovars, thereby reflecting predominant overlap of TnM and CM (Fisher’s exact test *P* < 0.0001) in Typhi and Paratyphi A, as well. It is noteworthy that out of 514 intact genes (i.e., devoid of any TnM) with CM in Typhi and Paratyphi A, 264 genes (51%) accumulated CM in both the serovars (Fig. 2b), thereby reconfirming the adaptive evolution of these serovars via convergent mutations as demonstrated by our earlier work (21). However, the frequencies of genes with CM genes within three different sets of TnM genes, (i) TnM in Typhi but not in Paratyphi A, (ii) TnM in Paratyphi A but not in Typhi, and (iii) TnM in both, were 58% (57 of 98), 68% (39 of 57), and 60% (3 of 5), suggesting no significant bias (Fisher’s exact test *P* > 0.2) of CM accumulation in one of these three sets of TnM genes, while the overlap of CM and TnM was found to be equally high (Fig. 2a). However, this does not rule out the possibility of adaptive accumulation of TnM in Typhi and Paratyphi A, and an understanding of this may reveal some key differences in selection pressures between these two otherwise parallelly evolving serovars.

### Robustness of limited overlap of Typhi and Paratyphi A TnM genes as evidenced by altering the size of analyzed sample set.

Although equivalent intraserovar diversity values and absence of any strong correlation between TnM gene frequencies with sample size across serovars hinted that the drastic difference of analyzed Typhi and Paratyphi A isolates (116 versus 6) would not much affect the frequency of TnM genes in those serovars, we altered the sample size from two different angles to test this further. First, instead of all 116 Typhi genomes, we randomly selected 5 sets of 6 Typhi genomes (Table S3 found at https://figshare.com/articles/dataset/Supplemental_Material_FOR_publication_pdf/19656858) to analyze with the remaining data set that included 6 Paratyphi A genomes, thereby having parity on the sample size for these two serovars. While the number of TnM genes for Paratyphi A and for both serovars remained the same (57 genes and 5 genes, respectively), the number of TnM genes for Typhi was reduced to 52 to 59 genes in the 5 sets (Fig. S1 found at https://figshare.com/articles/dataset/Supplemental_Material_FOR_publication_pdf/19656858), in contrast to 98 TnM genes found for 116 Typhi genomes. This range of TnM genes in sets of 6 Typhi isolates, being equivalent to the number detected for 6 Paratyphi isolates, also speaks to the minimal, if any, contribution of sequencing errors to the values of TnM genes found in either Typhi or Paratyphi A isolates analyzed.

Next, while keeping all available 116 completed genomes of Typhi, we added to our analysis 25 draft genomes of Paratyphi A (Table S4 found at https://figshare.com/articles/dataset/Supplemental_Material_FOR_publication_pdf/19656858), thereby increasing the sample size for this serovar a bit, since no more completely sequenced Paratyphi A genomes were available. We sorted all draft Paratyphi A genomes available in GenBank in descending order for the number of coding DNA sequences annotated and selected the top 25 isolates from the list, with a goal to maximize the availability of core genes detected based on 214 previously completely sequenced genomes. The findings from this new analysis (Fig. S2 found at https://figshare.com/articles/dataset/Supplemental_Material_FOR_publication_pdf/19656858) revealed only a few additional TnM genes for Paratyphi A (66 genes in contrast to 57 genes based on 6 isolates). However, the number of TnM genes overlapping in both serovars increased by only one. The gene *mdtQ* (encoding multidrug resistance outer membrane protein MdtQ), which earlier had TnM in 5 Typhi isolates (across two different allelic backgrounds via two independent truncation mutations), was found to accumulate TnM also in 4 draft genome isolates of Paratyphi A. Therefore, the number of TnM genes for Typhi moved to 97 from the total of 98 for 116 genomes (Fig. S2 found at https://figshare.com/articles/dataset/Supplemental_Material_FOR_publication_pdf/19656858). Although the number of TnM genes changed in Typhi and Paratyphi A separately with the change of sample size of either Typhi or Paratyphi A, we detected almost no change in the limited overlap of TnM genes in these serovars, reconfirming the presence of distinct selection pressures leading to the accumulation of TnM in these two serovars despite their adaptation to the same host species.

### Distinct functional groups represented by the TnM genes in Typhi versus Paratyphi A.

**FIG 3:**
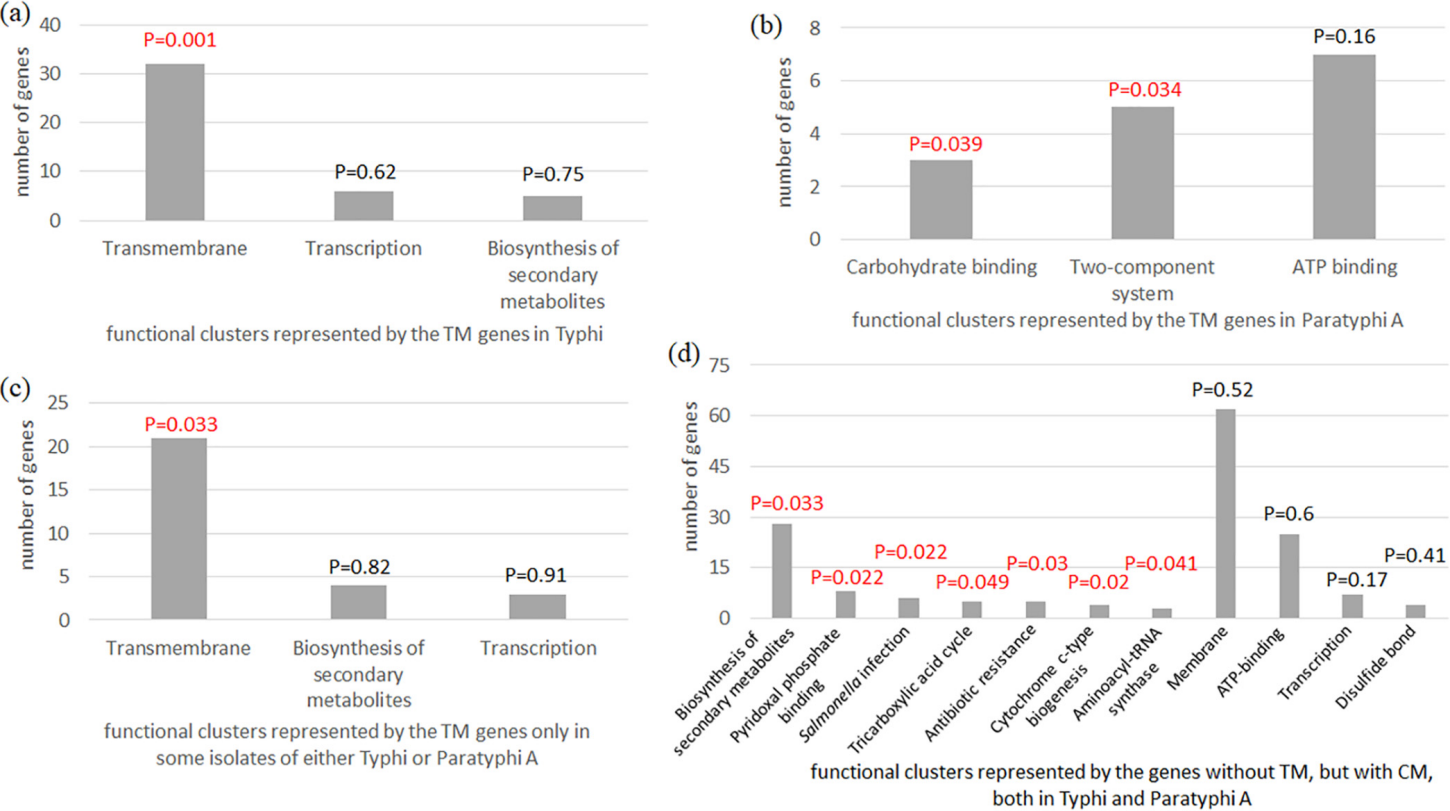
Functional annotation clustering and enrichment analysis of (a) TnM genes in Typhi (but not Paratyphi A), (b) TnM genes in Paratyphi A (but not Typhi), (c) TnM genes only in some isolates analyzed for either Typhi or Paratyphi A, and (d) nontruncated genes with CM in both Typhi and Paratyphi A. TnM and CM denote truncation mutations and convergent amino acid mutations, respectively. The enriched or overrepresented clusters are shown with *P* values in red (*P* < 0.05).

We next performed the functional classification and enrichment analysis for the TnM genes in Typhi and Paratyphi A separately. The set of 98 TnM genes in Typhi included 57 genes with CM in different serovar isolates and 41 genes without any CM. Interestingly, the TnM genes with and without CM hardly showed any difference in the list of protein functional clusters encoded by them, and only the transmembrane group of proteins emerged as the single overrepresented cluster in both subsets as well as in the entire set of 98 TnM genes (*P* = 0.001) of a total of three clusters detected in the analysis (Fig. 3a). On the other hand, in Paratyphi A, while the membrane or transmembrane protein clusters were present in the subsets of TnM genes with or without CM, the entire set of 57 TnM genes represented two overrepresented clusters (Fig. 3b): two-component system (*P* = 0.044), which comprised some of the membrane genes only, and carbohydrate-binding protein group (*P* = 0.039), which was not detected in Typhi. The set of 5 genes that accumulated TnM in all Typhi and Paratyphi A isolates included 3 hypothetical genes, while the remaining two encoded a nonspecific endonuclease and one MFS transporter family protein, thereby not allowing any enrichment analysis of this set.

Importantly, of 98 TnM genes in Typhi (that did not show TnM in Paratyphi A), there were 53 genes (54%) for which not all the isolates of Typhi serovar accumulated TnM (Table S2 found at https://figshare.com/articles/dataset/Supplemental_Material_FOR_publication_pdf/19656858). Of this set, 47 genes showed TnM in only 1 to 10 isolates of 116, while the remaining 6 genes had TnM in 71, 79, 93, 107, 113, and 114 isolates. In Paratyphi A, the number of such genes (not showing TnM in Typhi) was 15 (26%), of which 9 genes accumulated TnM only in 1 of 6 analyzed isolates of Paratyphi A serovar (Table S2 found at https://figshare.com/articles/dataset/Supplemental_Material_FOR_publication_pdf/19656858). Similarly, of all 98 TnM genes in Typhi, 50 genes (51%) did not show representation of all five STs (1, 2, 8, 2138, and 2209) that were present in the Typhi isolates analyzed (Table S2 found at https://figshare.com/articles/dataset/Supplemental_Material_FOR_publication_pdf/19656858). Interestingly, 26 (52%) of these genes had TnM only in ST2, with a slightly higher frequency in genes with CM (59%). While the Paratyphi A isolates analyzed were represented by two STs (85 and 129), the frequency of genes with TnM in either of these STs was 19% (Table S2 found at https://figshare.com/articles/dataset/Supplemental_Material_FOR_publication_pdf/19656858).

If the TnM accumulation would be due to reductive evolution linked to the host-restricted lifestyle of a given serovar, it is expected that the TnM genes would be found to accumulate such mutations in all analyzed isolates of that serovar, unlike the scenario represented by these 68 genes (53 for Typhi and 15 for Paratyphi A). Also, there were 61 genes where the TnM accumulated only in one or some STs, but not in all (50 for Typhi and 11 for Paratyphi A), while the ST2 in Typhi appeared to be the predominant clone to acquire the TnM (Table S2 found at https://figshare.com/articles/dataset/Supplemental_Material_FOR_publication_pdf/19656858). Therefore, we performed functional enrichment analysis exclusively for the set of 68 genes (Fig. 3c) to again reveal the transmembrane group as the single overrepresented cluster (*P* = 0.033). We anticipate that, in this set of genes (Table S2 found at https://figshare.com/articles/dataset/Supplemental_Material_FOR_publication_pdf/19656858), the TnM accumulated selectively in isolates of a given serovar were mediated by adaptive pressures, for which future functional studies are warranted.

We also wanted to compare the distribution and enrichment of functional clusters for the intact genes (i.e., without any TnM) accumulating CM in Typhi and Paratyphi A with the results obtained for the Typhi and Paratyphi sets of TnM genes. No overrepresented cluster was detected for the 149 genes with CM in Typhi (but not in Paratyphi A), while signal transduction histidine kinase-related protein family was the only overrepresented cluster (*P* = 0.015) in 101 genes with CM in Paratyphi A (but not in Typhi). In the set of 264 genes with CM in both Typhi and Paratyphi A, we found 11 prominent functional clusters with little overlap of genes among them (Fig. 3d), of which 7 clusters were overrepresented: tricarboxylic acid cycle (*P* = 0.049), pyridoxal phosphate binding (*P* = 0.022), antibiotic resistance (*P* = 0.03), biosynthesis of secondary metabolites (*P* = 0.033), cytochrome c-type biogenesis (*P* = 0.02), *Salmonella* infection (*P* = 0.022), and aminoacyl-tRNA synthetase (*P* = 0.041). While cytochrome c-type biogenesis and aminoacyl-tRNA synthetase clusters were subsets of membrane and ATP-binding clusters, respectively, both membrane (*P* = 0.52) and ATP-binding (*P* = 0.60) clusters emerged in the analysis as nonoverrepresented clusters, unlike what we detected for the TnM genes of Typhi and Paratyphi A, respectively. While the diversity of both overrepresented and nonoverrepresented clusters could be partially attributed to a higher number of genes being analyzed, the enrichment results clearly showed the distinct evolutionary forces to be in action for non-TnM genes with CM compared to the TnM genes with or without CM. Moreover, such distinction was maintained even between Typhi and Paratyphi TnM genes; while the former reflected enrichment of transmembrane family of proteins, the latter serovar showed enrichment primarily of carbohydrate-binding, ATP-binding, and two-component system proteins. This essentially demonstrates lack of any significant convergence in the accumulation of TnM in the core genes of Typhi and Paratyphi A, which is in contrast to the known convergent adaptive evolution of these two serovars via recombination, mutation, and pseudogene accumulation (20–22). Overall, such an observation presents another indirect piece of evidence that, in conjunction with the reductive evolution-mediated accumulation of TnM, a sizable fraction of genes in both Typhi and Paratyphi A have acquired TnM in response to distinct positive selection pressures not only in the specific serovar but differentially in specific isolates of a single serovar, possibly enabling them to gain some immediate advantages under particular environmental conditions.

### Inactivation of several transmembrane genes in Typhi potentially linked to increased virulence.

In Typhi, the single overrepresented cluster included 32 genes encoding the transmembrane proteins. Owing to the cellular location of this family of proteins, the prevalence of TnM in this functional cluster could be directly linked to the various selection pressures exerted by the host-immune system as well as various antibiotics. It is worth mentioning that, although we anticipate here that accumulated TnM would lead to gene inactivation affecting the evolution of virulence, directed functional assays are necessary to validate these possibilities, which could also generate new hypotheses.

Of the 32 genes in this cluster, 3 genes were found to belong to MFS (major facilitator superfamily) family proteins involved in the transport of arabinose (AraJ), putative nucleoside transport (YegT), and an inositol transporter (YaaU). Arabinose works as the only strong pentose repressor of the virulence genes present in the *Salmonella* pathogenicity island 1 (SPI-1). The repression mediated by l-arabinose is exerted at a single target (HilD) that consequently reduces the translocation of SPI-1 effector proteins to the epithelial cells and is shown to reduce the invasion of Salmonella *in vitro* (33). As a result, it is only reasonable to speculate that the accumulation of TnM in the *araJ* gene (encoding the protein to mediate arabinose transport) could lead to reduced arabinose uptake and subsequent increase in the virulence of the pathogen. A basic InterPro analysis showed that YegT belonged to the family of NupG nucleoside permease (34). Importantly, NupG and NupC have been shown to be responsible for the active transport of nucleosides to the bacterial cytoplasm, playing a role in the survival of the pathogen inside the host cells (35). Therefore, it is worth determining whether the inactivation of *yegT* gene would trigger any antivirulence mechanism and its impact on the pathogen. Certain pathogens, like various serovars of S. enterica, can survive completely on alternate sugars like *myo*-inositol (MI), as they have specialized pathways for MI metabolism (36). Inactivation of certain inositol transporters (YaaU) in Typhi possibly highlights the metabolic redundancy in the pathogen, the importance of which in its virulence mechanisms is yet to be understood.

In addition to MFS transporters, two efflux pump protein-encoding genes were also found to accumulate TnM, e.g., the resistance-nodulation-cell division (RND) family acridine efflux pump (encoded by *acrB*) and the multidrug efflux protein AcrF (encoded by *acrF*). AcrB not only plays a very crucial role in the efflux of several host-derived antimicrobial compounds like bile salts but also helps in the colonization and bacterial adaptation to the animal intestinal tracts. Mutants of *acrB* are found to have reduced efficiency in adhering, invading, and surviving in mouse monocyte macrophages, and they failed to persist in the host gastrointestinal tracts. This suggests that the functional AcrB might not be relevant at the initial stages of gut colonization, whereas the presence of AcrB is crucial for gastrointestinal persistence (37). Previous studies have reported that, unlike the loss of AcrB protein, the loss of efflux function of AcrB did not induce overexpression of other RND efflux pumps (38). On the other hand, a genome-wide transcriptional analysis revealed that the inactivation of *acrB* led to drastic increase in the expression of *ramA*, shown to be linked with multidrug resistance in S. enterica (39, 40). Therefore, it is important to experimentally validate the physiological impact of this TnM. In addition to the efflux pumps, an outer membrane efflux protein MdtQ (encoded by *yohG*) was also found to harbor premature stop codons. Essentially, inactivation of the function of this gene did not have an impact on the intrinsic level of resistance to antibiotics. However, it was found that the isolates lacking this gene showed a slightly higher susceptibility to puromycin, a protein translation inhibitor (41, 42). Interestingly, there were multiple independent accumulations of premature stop codon mutations at different positions in the MdtQ-encoding gene in our analyzed Typhi isolates, one in an allele represented by 4 isolates and another in an allele with a singleton, while the remaining 111 isolates had intact copies of the gene, thereby suggesting that potential selection pressures result in the inactivation of this gene only in specific isolates of Typhi.

Another TnM gene of Typhi, representing the transmembrane functional cluster, encoded a putative methyl-accepting chemotactic protein (MCP). Previous study on selection-driven gene inactivation in *Salmonella* reports the presence of premature stop codons in an MCP gene locus (*tsr*) (17). Inactivation of an MCP gene might partially impede motility and chemotaxis. This could also result in keeping bacteria more stationary when they otherwise could have easily moved along a concentration gradient. The loss of function of one MCP might help the bacteria to follow a weaker concentration gradient sensed by another MCP. In essence, it could be such that the bacteria become more adapted to responding to even weaker concentrations sensed by other MCPs (17). A recent study reported that HilD (a virulence regulator) positively regulates McpC (a methyl-accepting chemotactic protein), showing direct correlation to smooth swimming phenotype responsible for invasiveness in *Salmonella* Typhimurium (43). Therefore, it is possible that the accumulation of TnM in one MCP, as detected here, might help the bacteria to better respond to the cues sensed by other MCPs, including McpC, potentially resulting in increased virulence.

The outer membrane porin protein (OmpF) is a strong antigen that can induce both innate and adaptive immune responses (44). We detected TnM in the *ompF* gene of the Typhi isolates. Interestingly, another porin gene, *ompC*, encoding the macrophage recognition protein accumulated TnM in Typhimurium serovar isolates. Previous works show that both OmpF and OmpC, as recognized by the macrophages, participate in phagocytosis and trigger a signaling cascade that regulates the reactive oxygen species (ROS) response by the host and the phagolysosomal maturation (45). We anticipate that the absence of one of these important porins might contribute to a reduced host-immune response, where the macrophage recognizes only the functional porin to progress to phagocytosis and other downstream cellular responses. Due to the absence of other functional porin(s), the subsequent cellular responses might not be strong enough, and this, when combined with the effector responses of the *Salmonella* virulence factors, may facilitate better invasion, persistence, or survival of the pathogen inside the host cell environment.

### Overrepresented functional clusters for the TnM genes in Paratyphi A found to have important roles in virulence.

We detected three functional clusters to be overrepresented in two sets of Paratyphi A TnM genes: (i) carbohydrate binding and ATP binding (in the set of TnM genes that also accumulated CM in different serovars) and (ii) carbohydrate binding and two-component system (in the entire set of genes with TnM in Paratyphi A). No functional clusters were found to be overrepresented in the set of Paratyphi A TnM genes without CM. In the carbohydrate-binding functional cluster, three genes belonged to the family of hydrolases: *yicL*, *STM0018*, and *STM0041*. Certain protein domains, like the carbohydrate binding module (CBM), facilitate binding of these enzymes to the carbohydrate moieties prior to their hydrolytic activity. In pathogens like *Salmonella*, there are 48 enzymes spread over 21 different families of glycosyl hydrolases to overcome the carbohydrate barrier of our gut epithelium (46). Although the majority of reports on the virulence of *Salmonella* infections describe the process in light of type 3 secretion system (T3SS), in order to interact with the host gut epithelia, the pathogen first needs to degrade several layers of glycans and glycocalyx, which is facilitated by an array of glycosyl hydrolases (46). However, the roles of this diverse family of hydrolases still remain highly understudied despite its importance in *Salmonella* infections.

Three TnM genes (*sufC*, *mglA*, and *yhiH*) representing the ATP-binding functional cluster belonged to ATP-binding cassette (ABC) superfamily of transporters, while another (*mgtA*) belonged to P-type ATPase superfamily. Virulence in pathogens like *Salmonella* is governed by their uptake of different molecules. It is known that the uptake of metal ions, different sugars, etc. can contribute to the virulence of the bacteria, e.g., *sufABCD* and *feoABC* operons are responsible for the transport of iron in *Erwinia* spp. and *Salmonella* Typhimurium, respectively (47). Similarly, magnesium ions are also found to function as an extracellular signal directly controlling the PhoP/PhoQ (two-component system) virulence regulatory system in *Salmonella*, which in turn regulates the expression of a large number of virulence genes (48).

Two-component signaling systems regulate a wide range of physiological functions in bacteria, ranging from nutrient acquisition to endospore formation. In our analysis, 5 genes (*gltJ*, *uhpT*, *uhpC*, *uhpB*, and *dpiB*) belonging to this functional cluster were found to harbor TnM in Paratyphi A. UhpT functions as a hexose phosphate transport protein that is induced specifically by extracellular glucose-6-phosphate (Glu6P) and not by intracellular Glu6P levels formed during the metabolism of glucose and other carbon sources. The transcription of *uhpT* is tightly regulated by *uhpABC* genes located immediately upstream to the *uhpT* gene. It is found that the synergistic action of all three genes in the *uhp* operon is necessary for the expression of *uhpT*, and it supports a model in which the UhpB and UhpC proteins function as membrane-localized proteins to be able to sense extracellular Glu6P levels, thus converting UhpA to a form capable of activating *uhpT* transcription (49). Interestingly, the deletion mutant of *uhpT* showed inhibition of growth *in vitro* in both enteroinvasive Escherichia coli and S. enterica Typhimurium in the presence of Glu6P, although the same mutant did not exhibit any reduction in their ability for intracellular replication in Caco-2 cells (50). The inactivation of almost an entire operon (i.e., *uhpB* and *uhpC* of the *uhpABC* operon) and also the transporter protein *uhpT* indicates that, to survive and maintain the intracellular persistence, the pathogen would have to opt for other metabolic pathways to utilize alternate sources of energy, similar to what we discussed earlier for Typhi regarding the MI utilization. It is expected that future experimental studies would explore the functional roles of all these groups of proteins in *Salmonella* host cell adherence and invasion, thereby deciphering any impact of inactivation of these genes on *Salmonella* virulence.

### Conclusions.

It is well known that, owing to the host-restricted lifestyle, the systemically invasive pathogens of S. enterica, such as Typhi and Paratyphi A, lack the necessity of the functionality of many of their genes, thereby allowing pseudogenization of such genes followed by deletion over evolutionary time. This leads to significantly reduced genomes in such invasive serovars. However, if the reductive evolutionary dynamics have to explain the entire scenario of pseudogene accumulation via inactivating mutations in genes, we would expect the following things: (i) the serovars that infect and are restricted to the same single host species and are adapted to infect, and even coinfect, the same tissues should have strong overlap in the genes targeted by TnM (31), (ii) most of the TnM should be found in all analyzed isolates of the serovar, and (iii) it should be rare that such genes with TnM would accumulate potential adaptive mutations (such as convergent amino acid mutations) in isolates of other invasive and noninvasive serovars.

Importantly, our analysis of Typhi and Paratyphi TnM genes in the background of other invasive and noninvasive serovars demonstrates significant deviations from all three expectations stated above. We detected very limited overlap in the TnM genes, and also in the overrepresented functional groups of encoded proteins, for Typhi and Paratyphi A, two human-restricted systemically invasive serovars that were shown to exhibit evolutionary convergence via both recombination and mutational events (20, 21). Second, high frequency of genes with TnM in a specific serovar, either Typhi or Paratyphi A, was detected only in one or some, but not all, isolates of that serovar. This suggests that the acquisition of TnM in those isolates within a serovar might be offering some immediate fitness advantage in response to specific selection pressures. Finally, a significant proportion of all these TnM genes, not only in Typhi or Paratyphi A but overall, harbored convergent amino acid mutations in different serovars, and sometimes even in isolates of the same serovar. However, we would like to make a clear acknowledgment that these results suggest the adaptive accumulation of TnM in some genes only in conjunction with TnM in many other genes as neutral changes (sometimes under relaxed selection pressures) or as deleterious ones. Also, whether the potentially positively selected TnM would offer only some immediate fitness advantage but might be detrimental in the long run (especially those that were found to accumulate in one or a few isolates of Typhi or Paratyphi A), or would be fixed in the population over time, can be assessed only via larger population-level studies.

Altogether, this study presents, for the first time to our knowledge, a genome-wide comparative study of TnM in *Salmonella* Typhi and *S.* Paratyphi A serovars in search of adaptive evolutionary footprints. Our results strongly suggest that a considerable fraction of core genes accumulate TnM under selection pressures, in conjunction with others that result from reductive evolution. An important next step, which has not been considered in this study, could be to decipher whether or not the TnM and CM in Typhi and Paratyphi A would cluster in the mutational hot spots, such as those induced by DNA cytosine methyltransferase or Dcm (51), by DNA adenine methyltransferase or Dam (52), or by stress owing to DNA double-strand-breaks (53), although our previous study on Escherichia coli did not show any correlations between CM and mutational hot spots (27). Also, apart from the TnM and CM in protein-coding genes, the mutations in other important regions, such as specific promoter and regulatory sites, can have a drastic impact on the expression of associated genes, often leading to inactivation. Therefore, future in-depth studies, both bioinformatic with larger data sets and experimental with directed functional assays, are necessary to assess the role of such types of noncoding region mutations as well, in conjunction with the TnM and inactivating nonsynonymous mutations, to achieve a more comprehensive understanding of the extent and impact of gene inactivation in the virulence evolution of bacterial pathogens.

## MATERIALS AND METHODS

### Analysis of MLST, sequence diversity, and statistical significance.

Using MLST database for Salmonella enterica, we detected the STs of all 214 isolates representing Typhi, Paratyphi A, Gallinarum, Pullorum, and Typhimurium serovars, based on the internal fragments of seven housekeeping genes: *aroC*, *dnaN*, *hemD*, *hisD*, *purE*, *sucA*, and *thrA* (https://pubmlst.org/bigsdb?db=pubmlst_salmonella_seqdef). The nucleotide sequence diversity was calculated using DnaSP v6 (54). For statistical significance test, Fisher’s exact test two-tailed *P* value was obtained via GraphPad QuickCalcs (https://www.graphpad.com/quickcalcs/).

### Pan- and core-genome profiling of the protein-coding genes.

The pan- and core-genome profiling of 214 genomes was performed using PanCoreGen using the protein-coding gene annotations of all the completed genomes sequentially as references (55). We used a highly stringent cutoff value of 95% for both nucleotide sequence identity and sequence-length coverage to avoid any impact of nonhomologous recombination and horizontal gene transfer in the data set of core genes for subsequent analyses.

### Phylogenetic analysis of TnM and convergent mutations of core genes.

Maximum-likelihood-based phylogenetic reconstruction of each of the core genes was done using PAUP* as implemented in TimeZone software package (56, 57). Detection of probable recombinant genes was performed using PhiPack software package as implemented in TimeZone (58). While the convergent amino acid mutations were identified through zonal phylogeny-based analysis of nucleotide sequences using TimeZone, an in-house script in conjunction with TimeZone led us to detect the presence of TnM in specific isolates and serovars (59).

### Functional classification and enrichment analysis.

DAVID was used for functional annotation clustering for different sets of candidate genes with or without TnM (60). The classification stringency was set as “medium” for each analysis. The enriched or overrepresented functional categories designate the analyses showing a *P* value of <0.05.

### Data availability.

The data sets supporting the conclusions of this article are included within the article and as part of the supplemental material found at https://figshare.com/articles/dataset/Supplemental_Material_FOR_publication_pdf/19656858. All the data are fully available without any restrictions.
